# The effect of mindfulness interventions on stress in medical students: A systematic review and meta-analysis

**DOI:** 10.1371/journal.pone.0286387

**Published:** 2023-10-05

**Authors:** Edie L. Sperling, Jennifer M. Hulett, LeeAnne B. Sherwin, Sarah Thompson, B. Ann Bettencourt

**Affiliations:** 1 Medical Anatomical Sciences, College of Osteopathic Medicine of the Pacific–Northwest, Western University of Health Sciences, Lebanon, Oregon, United States of America; 2 Sinclair School of Nursing, University of Missouri, Columbia, Missouri, United States of America; 3 Ellis Fischel Cancer Center, University of Missouri, Columbia, Missouri, United States of America; 4 Department of Psychological Sciences, University of Missouri, Columbia, Missouri, United States of America; Universitat Wien, AUSTRIA

## Abstract

**Background:**

Medical students have high levels of stress, which is associated with higher incidents of burnout, depression, and suicide compared to age-matched peers. Mindfulness practices have been shown to reduce stress among medical students.

**Purpose:**

The purpose of this systematic review and meta-analysis was to examine if mindfulness interventions have an overall effect on stress outcomes in the high-stress population of medical students globally, particularly given the wide variety of interventions. Any intervention designed to promote mindfulness was included.

**Methods:**

A comprehensive literature search was completed to include multiple databases, ancestry, and hand-searching and 35 studies were included. Standardized mean difference effect sizes (ES) were synthesized across studies using a random-effects model for changes in stress levels in medical students ≥ 18. Moderator analyses were performed to explore variations in effects by participant and intervention characteristics.

**Results:**

Mindfulness interventions significantly improved stress among medical students in both the two-arm studies (*d* = 0.370, *k* = 19, *n* = 2,199, 95% *CI* 0.239–0.501, *p* < .001) and one-arm pre-post studies (*d* = 0.291, *k* = 30, *n* = 18 (two cohorts from Dyrbye et al), 95% *CI* 0.127–0.455, *p* = 0.001). Moderator analyses found trends in less hours and less required practice resulted in better improvement in stress.

**Conclusions:**

This study further confirms that despite a wide variety of mindfulness interventions for medical students around the world, they produce an overall small-to-moderate effect on stress reduction. Future research looking at the most effective protocols for high-stress medical students would be beneficial.

## Introduction

The onset of stress begins very early in medical training and continues to be high throughout medical school and residency [1, 2]. In a recent study, 27.2% of medical students had depression and 11.1% had suicidal ideation [3]. Despite lower baseline levels of suicidality, suicide rates are higher among medical students only months after beginning medical school compared to age-matched peers [3, 4]. Further research shows that 34% of medical students experience emotional exhaustion, 34% feel high levels of depersonalization towards others, and 46.6% feel low levels of a sense of personal accomplishment [5]. More than two-thirds of medical students report sleep deprivation, lack of control over their schedule, and lack of confidence in their knowledge and skills [6]. This alarming trend continues throughout the lengthy process of becoming a physician, suggesting medical training contributes to the emergence of mental and physical health conditions [4].

The negative effects of chronic stress are well known to cause physiologic and/or psychological illness and disease [7–16]. Mindfulness interventions have been consistently shown to reduce stress in the general population are relatively simple to implement, low-cost, have negligible risks, and require low time commitments [17]. A recent systematic review evaluating the cost-effectiveness of mindfulness found that mindfulness interventions were more effective and less expensive than standard care for individuals with depression, among other conditions [18]. Mindfulness has not been consistently shown to assist with stress reduction in medical students [19–22], although a recent systematic review of meta-analyses demonstrates mindfulness was more effective than passive controls in healthcare professionals/trainees and allied health students, but not better than active controls in allied health students [23]. Medical students were not examined separately [23]. Our meta-analysis aims to add to the evidence on mindfulness-based interventions among medical students internationally, to assess the range of interventions being utilized, to determine if certain intervention or participant characteristics changed the degree of effectiveness, and to compile best practices from international sources.

Mindfulness is generally intended to help practitioners focus on the present moment, noticing their thoughts and emotions without judgement [17]. There are many types of mindfulness practices, or ways to achieve mindfulness. Common types of mindfulness practices include seated or walking meditation, yoga, breathwork, and mantras, which can reduce anxiety and depression, improve emotional coping, and increase self-awareness [19–22]. Objective physiological measures have also provided support for mindfulness, including decreased circulating levels of C-reactive protein and proinflammatory cytokines [24–27]; increased telomere length and telomerase activity [24, 25]; as well as reduced proinflammatory cytokines in those with depression and anxiety [28]. In medical students specifically, stress-related epigenetic expression of *SLC6A4*, a serotonin transporter gene, has been found to increase after mindfulness interventions [29], while serum cortisol decreased [30].

Past reviews and meta-analyses on mindfulness interventions have conflicting findings which this meta-analysis aims to correct. Some systematic reviews and meta-analyses have found mixed or no impact of mindfulness interventions on stress-related outcomes among medical students [19–22, 31]; while others found a reduction in stress outcomes [20]. Past reviews did not search gray literature [19] which this one did in order to reduce publication bias. One review did not assess stress specifically among medical students, limiting generalizability [21]. This meta-analysis corrects for these issues and adds 17 new studies for analysis. Table 1 lists the previous and current reviews and which articles are included in each per the PRISMA guidelines (see Table 1). We also explore topics previous reviews have not considered: do intervention effects vary based on country of study, year of students in school, duration of mindfulness practice, or with study design?

**Table 1:** Comparison with recent meta-analyses.

	**1**	**2**	**3**	**4**	**5**	**6**	**7**	**8**	**9**	**10**	**11**	**12**	**13**	**14**	**15**	**16**	**17**	**18**	**19**	**20**	**21**	**22**	**23**	**24**	**25**	**26**	**27**	**28**	**29**	**30**	**31**	**32**	**33**	**34**	**35**	**36**	**37**
Daya & Hearn																																					
Haithaissard et al.																																					
McConnville et al.																																					
Witt et al.																																					
Yogeswaran et al.																																					
This review, Sperling et al																																					

Bond et al., 2013 [48]

Chen et al., 2016: not included due to lack of data [87]

Danilewitz et al., 2016 [42]

Garneau et al., 2013 [63]

Phang (Kar) et al., 2015 [44]

Warnecke et al., 2011 [45]

Damião Neto et al., 2019 [41]

Erogul et al., 2014 [43]

Yang et al., 2018 [46]

Zheng et al., 2015: not included due to population being traditional Chinese medicine students [88]

Finkelstein et al., 2007 [51]

Keng et al., 2015 [70]

Paholpak et al. [62]

Rosenzweig et al. [56]

Bughi et al., 2006 [49]

Greeson et al., 2015 [52]

Hassed et al., 2009 [53]

Simard & Henry, 2009 [68]

Slavin et al., 2014 [57]

Moore et al., 2020 [71]

Bansal et al, 2013 [60]

Dyrbye et al., 2017 [50]

Kakoschke et al., 2021 [54]

Kemper & Yun, 2014 [72]

Kraemer et al., 2016 [64]

Lampe & Müller-Hilke, 2021 [33]

MacLean et al., 2020 [61]

Motz et al., 2012 [65]

Óro et al. (2021) [55]

Parshad et al., 2011 [66]

Prasad et al., 2016 [67]

Stoffel et al., 2019 [29]

Turakitwanakan et al., 2013 [30]

van Dijk et al., 2017 [58]

Waechter et al., 2021 [47]

Williams et al., 2020 [69]

Zúñiga et al. (2022) [59]

## Methods

This systematic review and meta-analysis followed the Preferred Reporting Items for Systematic Reviews and MetaAnalyses (PRISMA) guidelines [32].

### Inclusion & exclusion criteria

**Fig 1:**
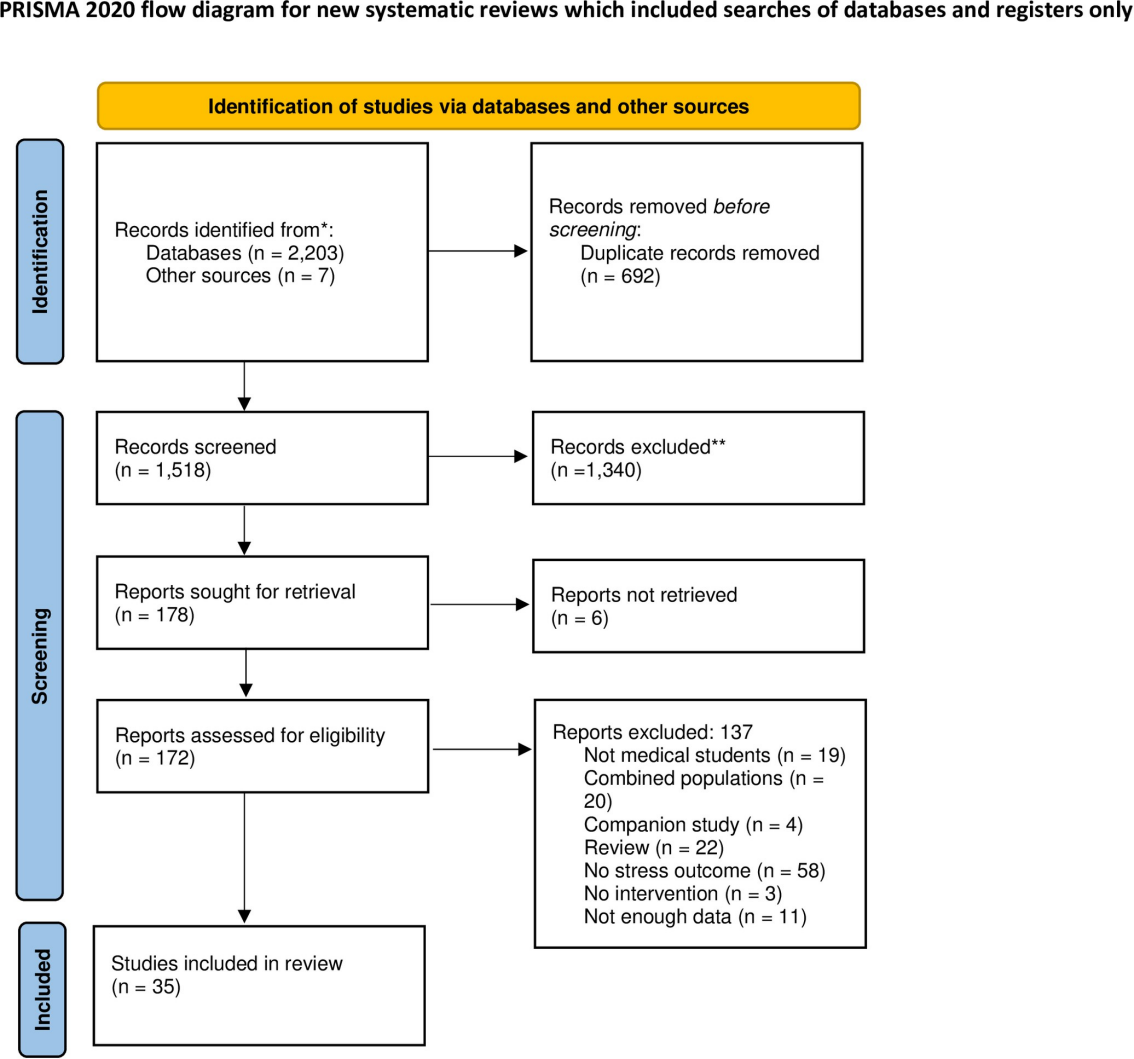
PRISMA flow diagram for study selection.

Inclusion criteria for the systematic review were (1) mindfulness component inclusive of sitting meditation, walking meditation, breathing exercises, body scanning, yoga, etc.; (2) medical students, including programs training medical doctors (MD), doctors of osteopathy (DO), or international equivalent (Bachelor of Medicine, Bachelor of surgery (MBBS), etc); (3) primary study of either single-arm pre/post design, randomized controlled trial (RCT), or two-arm non-randomized design; (4) written in or translated into English; (5) 18 years of age or older; and (6) reporting at least one stress outcome measure. Studies must have reported sufficient data to calculate effect sizes. Corresponding authors from three studies were emailed to request data appropriate for the meta-analysis, and one response was received, which was then able to be included in the meta-analysis [33]. Eligible stress outcomes were determined using the definition of stress from the National Cancer Institute [34]. Examples of stress outcomes include The Perceived Stress Scale (PSS) and the stress subscale from the Depression, Anxiety, Stress Scale (DASS; see Table 2 for all outcome measures). Burnout, anxiety, depression, compassion, compassion fatigue, perfectionism, and coping are separately defined; therefore, these outcomes were not included. No restrictions were placed on study location or date. Data of physiological measures of stress were collected, coded, and included in the appropriate meta-analysis alongside the self-reported measures if available (Fig 1). Studies were excluded if they had combined populations for which separate data were not available (e.g. medical and dental students), or were not primary studies (e.g. systematic reviews).

**Table 2:** Description and effect sizes of studies in the systematic review and meta-analysis.

Study/ Location	Design^a^	Intervention	Sample Characteristics	Outcome Measure/Results^b^
Bansal et al. (2013) India	One arm pre/post	Yoga, meditation Duration: 4 weeks Frequency: Daily for 45 minutes Total number of sessions: ~30	Age range: 18–23; 55.5% female N = 82	GHQ-28 d = 0.338
Bond et al. (2013) United States	One arm pre/post	“Embodied Health:” yoga, meditation, didactics Duration: 11 weeks Frequency: Once per week for 90 minutes Total number of sessions: 11	N = 27	PSS d = 0.081
Bughi et al. (2006) United States	One arm pre/post	Duration: 1 month Frequency: One session Total number of sessions: 1	Mean age 27.4 (3.2); 45.2% female; 51% white/42% Asian/7% Hispanic and Black N = 104	GWBS d = 0.414
Damião Neto et al. (2019) Brazil	Randomized controlled trial	Modified MBSR: didactics, body scanning, nonjudgmental listening, breathing exercises, visualization, and focus of attention Duration: 6 weeks Frequency: Once per week for 2 hours Total number of sessions: 6	Tx mean age 18.87 (1.81); 51.4% female; 65.7% white/34.4% non-white; N = 141	Stress subscale DASS-21 d = 0.100
Danilewitz et al. (2016) Canada	Randomized controlled trial	Mindfulness Meditation Program: stress-management, self-acceptance, perfectionism, loving kindness Duration: 8 weeks Frequency: Once per week for 75 minutes Total number of sessions: 8	N = 29	Stress subscale DASS-21 d = 0.336
Dyrbye et al. 2017 United States	One arm pre/post	Stress Management and Resilience Training (SMART): intentional awareness, attention, and attitude Duration: ~32 weeks? Frequency: unknown Total number of sessions: 2014:8/2015:9	2014 cohort: 58.3% female N = 44 2015 cohort: 59.1% female N = 22	PSS 2014 cohort: d = -0.754 2015 cohort: d = -0.510
Erogul et al. (2014) United States	Randomized controlled trial	Abridged MBSR: didactics, meditation, body scan, breathing exercises Duration: 8 weeks for avg. 75 minutes Frequency: Once per week Total number of sessions: 9	Tx mean age 23.6 (1.9); 42.9% female N = 59	PSS d = 0.610
Finkelstein et al. (2007) United States	Two-arm non-randomized	“Mind-Body Medicine” elective: didactics, meditation Duration: 10 weeks Frequency: Once per week for avg. 2 hours Total number of sessions: 10	Tx mean age 25.2 (2.2); 77.3% female N = 51	PSMS d = 0.027
Garneau et al. (2013) Canada	One arm pre/post	“Mindful Medical Practice: elective: meditation, yoga Duration: 4 weeks Frequency: Twice per week, 150 minutes Total number of sessions: 9	Tx mean age 26; 74% female; 50% white/50% non-white N = 58	PSS d = 0.301
Greeson et al. (2015) United States	One arm pre/post	“Mind-Body Medicine: Skill Building and Self-Care Workshop”: didactics, meditation Duration: 4 weeks Frequency: Once per week, 90 minutes Total number of sessions: 4	Total sample: 65.9% female N = 44	PSS d = 1.191
Hassed et al. (2009) Australia	One arm pre/post	“Health Enhancement Program” and “ESSENCE lifestyle program” Duration: 8 weeks Frequency: Once per week, 3-hour lectures, 2-hour tutorials Total number of sessions: 8	Total sample mean age 18.77 (1.10); 57.43% female N = 148	GSI d = 0.150
Kakoschke et al. (2021) Australia	One arm pre/post	“Health Enhancement Program” Duration: 5 weeks Frequency: Once per week, 2 hours Total number of sessions: 5	Total sample mean age 18.6; 60% female N = 205	PSS d = 0.273
Kemper & Yun (2014) United States	One arm pre/post	Hybrid online MBSR Duration: 8 weeks Frequency: Once per week, 1 hour Total number of sessions: 8	Age 29; “about half male” N = 4	PSS d = 0.186
Keng et al. (2015) Malaysia	Two-arm non-randomized	Mindful-Gym DVD Duration: 4 weeks Frequency: Once per week, 3 hours Total number of sessions: 4	Tx mean age 22.4 (0.55); 61.2% female N = 134	PSS d = 0.448
Kraemer et al. (2016) United States	Two-arm non-randomized	Mind-Body Medicine Skills course: biofeedback, guided imagery, relaxation, meditation styles, breathing, autogenic training Duration: 11 weeks Frequency: Once per week, 2 hours Total number of sessions: 11	Tx mean age 23.63 (1.76); 63.47% female; full sample 65.4% white/5.8% Black/15.4% Asian/5.8% other N = 52	PSS d = -0.011
Lampe & Müller-Hilke (2021) Germany	Two-arm non-randomized	Modified MBSR Duration: ~12 weeks Frequency: Every other week, 2 hours Total number of sessions: 6	N = 108	PSS d = 0.013
MacLean et al. (2020) Canada	Two-arm non-randomized	Mindfulness curriculum Duration: 3 years Frequency: unknown Total number of sessions: 9	N = 132	PSS d = 0.611
Moore et al. (2020) Australia	One arm pre/post	“Mindfulness Training Program”: emailed audiovisual link Duration: 8 weeks Frequency: 10 minutes weekly Total number of sessions: 8	Total sample mean age 26.7 (3.9); 80.9% female; 76.6% white/8.5% Asian/2.1% Latin American; 12.8% other N = 47	PSS d = 0.391
Motz et al. (2012) United States	One arm pre/post	Mind-Body Medicine Skills course: meditation, autogenic training, guided imagery, movement Duration: unknown Frequency: unknown Total number of sessions: unknown	65.27% female N = 72	PSS d = 0.486
Óro et al. (2021) Spain	Two-arm non-randomized	Modified MBSR Duration: 16 weeks Frequency: Every other week, 2 hours Total number of sessions: 8	N = 143	PSS d = 0.442
Paholpak et al. (2012) Thailand	Randomized controlled trial	Buddhist Anapanasati Meditation Duration: 28 days Frequency: Daily, 20 minutes Total number of sessions: 28	Tx mean age 23.43 (2.596); 50% female N = 58	GSI d = 0.863
Parshad et al. (2011) Jamaica	One arm pre/post	Yoga Duration: 6 weeks Frequency: Once per week, 1 hour Total number of sessions: 6	Total sample mean age 21.3 (2.6); 89.06% female N = 64	Biomarkers: SBP, DBP, MAP, HR, IBI, LVET, SV, CO, TPR, Zao, Cwk
Phang et al. (sometimes listed as Kar; 2015) Malaysia	Randomized controlled trial	Mindful-Gym DVD Duration: 5 weeks Frequency: unknown Total number of sessions: 5	Tx mean age 20.91 (1.15); 76% female N = 75	PSS d = 0.706
Prasad et al. (2016) United States	One arm pre/post	Hatha yoga and meditation Duration: 6 weeks Frequency: Twice per week, 1 hour Total number of sessions: 12	Total sample median age 28; 48.14% female; 48% white/ 11.1% Asian/ 7.4% Black/22% other N = 27	PSS d = 0.608
Rosenzweig et al. (2003) United States	Two-arm non-randomized	MBSR Duration: 10 weeks Frequency: Once per week, avg 90 minutes Total number of sessions: 10	N = 140	PMS d = 0.144
Simard & Henry (2009) Canada	One arm pre/post	Hatha yoga and meditation Duration: 16 weeks Frequency: Twice per week, 1 hour Total number of sessions: 32	Total sample mean age 22 (2.16); 56% female N = 14	PSS, GHQ-12 d = 1.376
Slavin et al. (2014) United States	Two-arm non-randomized	Curricular: resilience, mindfulness Duration: 1 semester Frequency: unknown Total number of sessions: 6	N = 525 Note: End of Year 1 as outcomes. 2011–12 control group data (before curriculum was implemented), 2015 data used as intervention group	PSS d = 0.835
Stoffel et al. (2019) Germany	Two-arm non-randomized	Mindfulness Based Intervention Duration: 3 months Frequency: Every other week, 3 hours Total number of sessions: 6	Tx mean age 20.79 (1.57); 64% female N = 74	*SLC6A4 DNAm* d = 0.863
Turakitwanakan et al. (2013) Thailand	One arm pre/post	Mindfulness meditation courses at Sunanthavanaram temple Duration: 4 days Frequency: Daily, 4 hours Total number of sessions: 4	Total sample mean age 19.1 (0.55); 66.67% female N = 30	GHQ-28, serum cortisol d = 0.555 combined (d = 0.312 self-report, d = 0.798 cortisol)
van Dijk et al. (2017) Netherlands	Randomized controlled trial	Modified MBSR Duration: 8 or 10 weeks (unclear) Frequency: Once per week, 2 hours Total number of sessions: 8 or 10 (unclear)	Tx mean age 23.7 (1.91); 60% female N = 167	GSI d = 0.185
Waechter et al. 2021 Grenada	Randomized controlled trial	Three-step process from Tibetan teacher Duration: 12 weeks Frequency: Twice per week, 1 hour Total number of sessions: 24	N = 42	PSS d = 0.076 combined (Mindfulness d = 0.500, yoga d = -0.107)
Warnecke et al. (2011) Australia	Randomized controlled trial	CD of guided mindfulness Duration: 8 weeks Frequency: Daily, 30 minutes Total number of sessions: 56	Tx mean age 23.4 (2.1); 74.2% female N = 31	PSS, stress subscale DASS-21 d = 0.612
Williams et al. (2020) United States	One arm pre/post	“Promoting Resilience in Medicine (PRIMe)” Duration: 11 weeks Frequency: Once per week, 2 hours Total number of sessions: 11	70.8% female N = 24	PSS d = 0.037
Yang et al. (2018) United States	Randomized controlled trial	Mobile phone app audio-guided meditation Duration: 30 days Frequency: Daily, 10 min for 10 days, 15 mins next 15 days, 20 mins for final 5 days Total number of sessions: 30	46.6% white/6.8% Black/25% Asian/5.7% Latinx/5.7% other N = 80	PSS d = 0.327
Zúñiga et al. (2022) Chile	One arm pre/post	Mandatory mindfulness course: burnout, emotional regulation, positive relationships, self-compassion, etc. Duration: 8 weeks Frequency: Once per week Total number of sessions: 8	48.8% female N = 102	PSS d = 0.461

^a^ Study Design: RCTs and two-arm non-randomized trials; one arm pre/post was not included. Note that a separate meta-analysis was run for the one-arm studies to obtain the effect sizes. They are not presented elsewhere, nor were they included in the meta-analysis.

^b^Outcome measure acronyms: General Well-Being Scale (GWBS)—Positive Well Being subscale; Depression, Anxiety and Stress Scale (DASS 21)—Stress subscale; Perceived Stress Scale (PSS); Global Severity Index (GSI); the Profile of Mood States (PMS)—Tension-Anxiety subscale; General Health Questionnaire-28 (GHQ-28); and the Perceived Stress of Medical School (PSMS) scale. Parshad et al (2011) used systolic blood pressure (SBP), diastolic blood pressure (DBP), mean arterial pressure (MAP), heart rate (HR), interbeat interval (IBI), left ventricular ejection time (LVET), stroke volume (SV), cardiac output (CO), total peripheral resistance (TPR), ascending aortic characteristic impedance (Zao), and total arterial compliance (Cwk).

### Search strategies

A research librarian was consulted to maximize the quality of the search. English language filters were used. The search was carried out between February 3 and May 4, 2022. The following 12 databases were searched: EBSCOhost (inclusive of Academic Search Elite; Alt Healthwatch; Business Source Elite; CINAHL; eBook Collection; ERIC; Funk & Wagnalls New World Encyclopedia; Health Source–Consumer Edition; Health Source–Nursing/Academic Edition; Library, Information Science & Technology Abstracts; MAS Ultra–School Edition; Military & Government Collection; Newspaper Source; Primary Search; OpenDissertations; APA PsycInfo; MAS Reference eBook Collection; Primary Search Reference eBook Collection; and MEDLINE); EBSCO Discovery, in the categories Complementary & Alternative Medicine, Health & Medicine, Nursing & Allied Health, Psychology, and Public Health; PubMed; Trip; Web of Science; Sociological Abstracts; SveMed+; Education Full Text; and Education Index Retrospective (available titles between 1923–1983). EBSCOhost and PubMed also search unpublished dissertations and conference abstracts. Publication Finder was searched for the following journals: Journal of Mind and Medical Sciences, International Journal of Medical Students, and Mindfulness, which were chosen due to the high probability that they might contain research on mindfulness in medical students. Research registries were searched including ClinicalTrials.gov and ISRCTN (condition categories Mental & Behavioral Disorders).

A structured search strategy was used, beginning with Boolean terms. If needed, these were then refined to MeSH terms. A Title/Abstract search was then done. Search terms included mindfulness, meditation, yoga, and medical students (see S1 Table.). The number of duplicates, irrelevant articles, non-English language articles, and books were recorded for reproducibility. A bibliographic search was done on six relevant published reviews [19–22, 35, 36]. Finally, hand searching strategies were used. The articles collected from the database searches were compared to see if there were common, frequently occurring journal names, which were then searched; these included the BMC Medical Education, Education in Medicine Journal, Medical Education Online, and Wisconsin Medical Journal. The search range for the hand search was set for 2014–2022. This date range was chosen due to when the topic of mindfulness in medical students was observed to become common: EBSCOhost and PubMed databases did not return more than two articles per year between 1977–2012, and only four articles in 2013. Article retrieval numbers increased to ten or more from 2014 onward.

### Data management

Zotero and Excel were used for data management. An Excel spreadsheet was used for tracking the search source, date, and terms.

### Data extraction and coding

A comprehensive codebook was developed per guidelines in Cooper [37]. The codebook draft was blind-reviewed and refined by two researchers. A coder trained in meta-analytic methods extracted data from eligible studies. Categories of variables were study identification, basic information (research design, funding, source of article), population characteristics and demographics (program type, location, mean age, gender, race/ethnicity), reliability and validity (blinding, attrition reporting, randomization), intervention details (name, length of intervention, duration of sessions, total sessions, location of sessions, delivery mode), outcomes (name, self-reported or physiologic, time point, direction of effect for outcome measure), and results (sample size, raw means, standards deviations, standard error, difference in means, and direction of effect for the intervention). The timepoints chosen for coding were the pre-intervention measures and the post-measure closest to the final day of the study, to provide consistency; most studies did not report other follow-up time points.

### Data analysis

Data analysis was conducted using Comprehensive Meta-Analysis Software (CMA). A random effects model was chosen *a priori* to account for expected heterogeneity among studies. Eligible studies included participants and interventions with a wide variety of characteristics, necessitating the use of the random effects model, which accounts for between-study and within-study variance [38].

**Fig 2:**
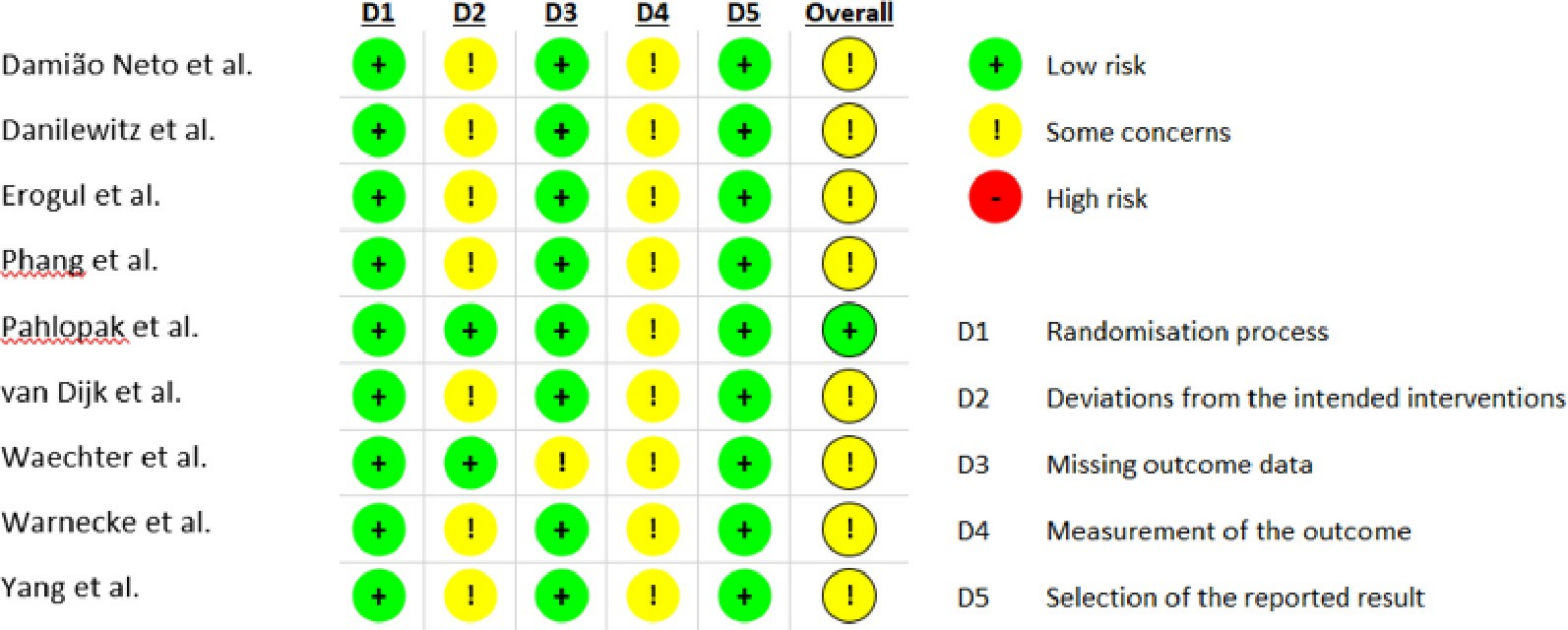
Risk of bias using RoB 2: A revised tool for assessing risk of bias in randomized trials for the RCT studies included in the meta-analysis [77].

Standardized mean difference effect sizes (ES, Cohen’s *d*) were calculated in CMA. Most were done by subtracting the mean of the control group from the mean of the treatment group and dividing by the pooled standard deviation (SD); two studies required use of difference in means and standard deviation; and two studies required use of difference in means and confidence intervals. Cohen’s *d* was chosen because of recent research showing Hedges’ *g* can result in more biased meta-estimation than Cohen’s *d* [39]. A positive ES is indicative of improved stress outcome measures and decreased stress, while a negative ES is indicative of worsened stress outcome measures and increased stress among medical students. In the one case where more than one self-reported stress outcome measure was used, they were combined in CMA by taking the average of the means [38]. Effect sizes were classified as small (≤ 0.20), medium (= 0.50), or large (≥ 0.80) [32]. Heterogeneity of the effects were assessed using Cochrane’s Q, I^2^, and T. Subgroup analysis and meta-regression were used to assess possible moderating effects of dichotomous or continuous variables, respectively. Cochrane’s Risk of Bias tool version 2 (RoB2) was used to evaluate the risk of bias across studies (Fig 2) [40].

## Results

The comprehensive database search identified 2,203 potential studies. A bibliography search and hand searching of journals identified 7 more potential studies. After removal of duplicates, 1,518 citations were screened for inclusion. The PRISMA flow diagram for study selection is shown in Fig 1 [32]. The 35 studies selected for inclusion in this systematic review are described in Table 2. The total sample size across the data sets included in the meta-analysis is 2,199 (intervention groups *n* = 954; control groups *n* = 1,245).

### Study and participant characteristics

The date of study publications ranged from 2003–2022. Nine of the studies were RCTs, and nine were non-randomized studies with separate intervention and control groups, with the remaining 17 single-arm, pre-post design. The studies included 14 (*k =* 15) conducted in the United States and 21 (*k =* 33) conducted in international medical programs. A total of 12 countries were represented outside of the U.S.: Australia (n = 4), Brazil (n = 1), Canada (n = 4), Chile (n = 1), Germany (n = 2), Grenada (n = 1), India (n = 1), Jamaica (n = 1), Malaysia (n = 2), the Netherlands (n = 1), Spain (n = 1), and Thailand (n = 2). The mean age of participants ranged from 18.87 to 25.2. Female participants made up between 47% to 77.3% of the samples in the studies. Nine of the 35 studies reported data on race or ethnicity. Study characteristics can be found in Table 2.

### Risk of bias

There were some concerns of risk of bias in the RCTs of this meta-analysis as evaluated with the RoB2 (Fig 2) [40]. One important risk of bias was due to non-adherence or possible non-adherence (i.e. not reported) of participants to the study protocols, which could have affected study outcomes [41–45]. Only four studies reported protocol adherence; two reported an average attendance of less than 50% [42, 46]. One study had a high drop-out rate (29%) and thus there was bias in the direction of the experimental hypothesis; as such it was coded as ‘some concern of bias.’ [47] Secondly, all studies had self-reported outcome measures, which increases risk of bias, and were subsequently coded as having ‘some concerns.’

### Intervention characteristics

To understand the diversity of interventions, articles were combed for intervention characteristics. Of the 35 eligible studies for systematic review, there were 18 different mindfulness intervention protocols used, demonstrating the expected heterogeneity as programs are modified for time, convenience, and resource availability. Many universities developed their own programs, some which are listed as mandatory curriculum, some elective. There was wide variety in how much mindfulness training students received; the duration of the intervention varied from 1 week to 16 weeks, and the frequency of sessions from every other week to three times per day. The durations of the individual sessions varied from approximately 2 minutes to 4 hours, and the total number of sessions from 1 to 56.

Meditation was included in all interventions. Many interventions also had didactic instruction on the theories behind mindfulness and mind-body medicine, or principles of neuroscience such as neuroplasticity [33, 41–44, 48–59]. Breathing exercises and/or breath awareness were also commonly taught [33, 41, 43, 49, 56, 60–63], as well as body scanning and visualization [33, 41, 44, 49, 56, 61, 64]. Yoga was part of many of the interventions [33, 47–49, 56, 60, 65–68]. Three reported instructing students in walking meditation [49, 56, 63], and two described instruction in self-acceptance or gratitude [42, 44]. Several described an educational intervention on emotional reactivity and non-judgement [41, 44, 53, 59]. Three studies reported a journaling component [54, 65, 69]. Two described instruction on the Buddhist principle of loving-kindness [42, 63]. Two reported instructions on biofeedback [64, 69], and one had communication exercises [61].

Several interventions were delivered by distance learning, including through DVD [44, 70], CD [45], mobile app [46] and online content [71]. Three studies had a hybrid format with both asynchronous and in-person components [55, 59, 72]. Many programs required after-hours mindfulness or meditation practice [33, 49, 51, 54–56, 62, 63, 71].

### Outcome measures

Nine different stress outcome measures were represented in all coded studies. The majority of studies (n = 23) used the Perceived Stress Scale (PSS), but the meta-analysis included a total of five different self-reported outcome measures (see Table 2). Warnecke et al. qualified for the meta-analysis and had two self-reported stress outcome measures, the PSS and the stress subscale of the DASS-21, which were combined in CMA by averaging the effect sizes per standard procedure. Dyrbye et al. had two cohorts, from 2014 and 2015; these were treated as separate samples [50]. Waechter et al. had three intervention groups, of which both the mindfulness and yoga groups was used in the meta-analysis [47] with a composite variable calculated by CMA.

Three studies investigated biomarkers instead of or in addition to self-reported outcome measures. Parshad et al. measured eleven cardiovascular biomarkers in healthy medical students before and after six weekly 1-hour yoga sessions and found statistically significant improvement in six of the eleven markers [66]. Stoffel et al. measured stress-related epigenetic expression of *SLC6A4*, a serotonin transporter gene, and found it increased after a 3-month mindfulness intervention (*p* < .0001) [29]; self-reported measures lacked enough detail to be included in the meta-analysis. Turakitwanakan et al. measured serum cortisol as a biomarker of stress, and found lower levels of serum cortisol after a four-day meditation retreat (*p* < .05); this study also had a self-reported stress outcome measure which also lacked sufficient information to be included in the meta-analysis [30].

### Summary effect sizes

**Fig 3:**
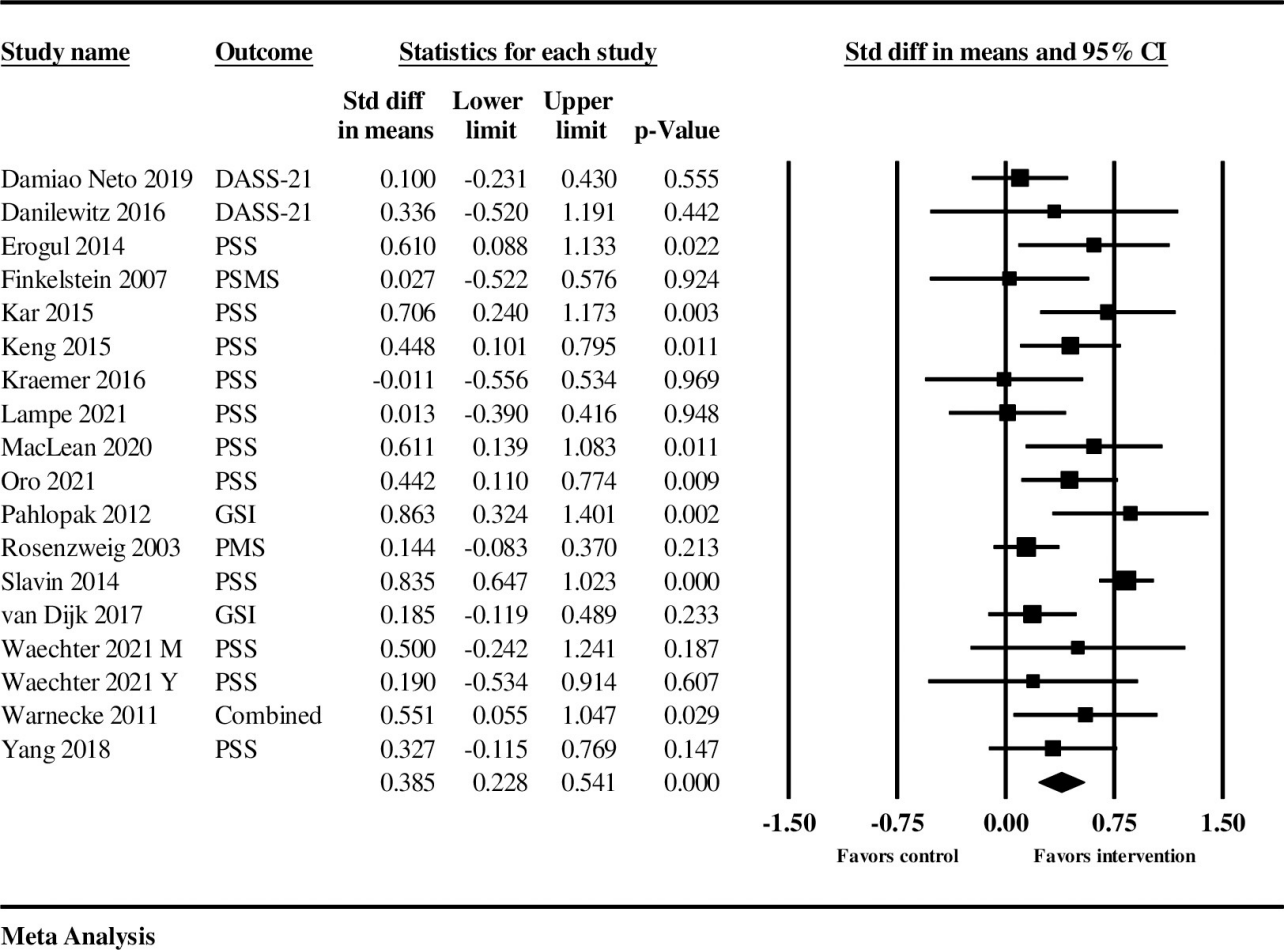
Summary statistics and forest plot with weighted means indicating the effect size, for the two-arm and RCTs in the meta-analysis.

Two separate meta-analyses were done, one for two-arm studies including RCTs, and one for one-arm pre-post studies, so that all effect sizes could be observed (see Table 2). The mindfulness interventions from the two-arm studies (*k* = 19, *n* = 18) had a statistically significant, small to moderate positive effect on reducing stress outcomes in medical students from pretest to posttest, compared to the controls (*d =* 0.370; 95% *CI* 0.239–0.501, *p* < .001) (Fig 3). Effect sizes ranged from -0.011 to 0.863 with a positive effect size indicating decreased stress and a negative effect size indicating increased stress following mindfulness interventions (Table 2 and Fig 3). As expected, there was statistically significant heterogeneity of effects noted across studies (*Q* = 31.947, *I*^*2*^ = 46.786%, *T* = 0.181, *p* < .001).

**Fig 4:**
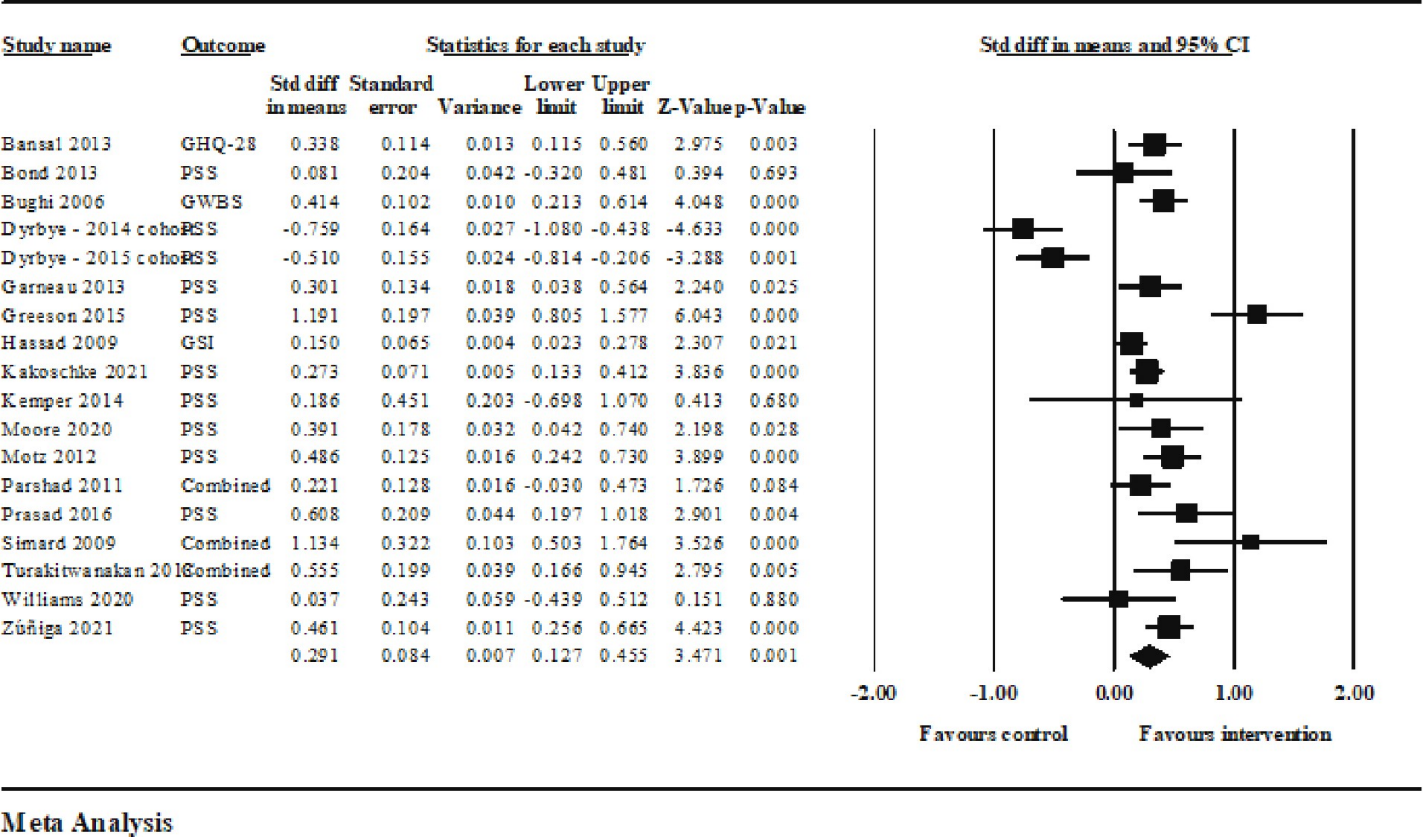
Summary statistics and forest plot with weighted means indicating the effect size, for the one-arm studies.

The mindfulness interventions in the one-arm studies (*k* = 30, *n* = 18) also had a statistically significant, small to moderate positive effect on reducing stress outcomes in medical students from pretest to posttest (*d* = 0.291, 95% *CI* 0.127–0.455, *p* = 0.001); Table 2 and Fig 4. (Note the *n* = 18 includes the two cohorts from Dyrbye et al., 2017, which had separate information from two cohorts and thus were produced separate effect sizes). Effect sizes ranged from -0.759 to 1.134 (Table 2, Fig 4). There was statistically significant heterogeneity of effects in this meta-analysis, also as expected (*Q* = 112.978, *I2* = 84.953%, *T* = 0.310, *p =* 0.001).

Publication bias was assessed using three methods: the ‘p-uniform’ test [73], funnel plots and [74], and Egger’s regression intercept [75]. Multiple methods were used in order to provide more confidence in our assessment of publication bias. The ‘p-uniform’ test was completed in R statistical software with the assistance of an expert in R; the funnel plots and Egger’s regression intercept were completed in CMA. The p-uniform test provides a meta-analytic point estimate corrected for publication bias [73]. It indicates that if there is no publication bias between affirmative (i.e., significant and positive) and non-affirmative (i.e., nonsignificant or negative) studies, the corrected meta-analytic point estimate for the two-arm studies would be 0.37 (95% CI [0.23–0.51]) and 0.28 (95% CI [0.07–0.49) for the one-arm studies [73]. Reassessment of both data sets was done with removal of potentially biased studies; for the one-arm meta-analysis this did not change results [64], and for the two-arm meta-analysis, there was less publication bias with the removal of one study with a large negative effect [50]. The p-uniform test also produces funnel plots, which indicated the meta-analyses for both groups of studies were robust to publication bias [73]; all of the data output from R, including the p-uniform funnel plots, are freely accessible in the data repository.

**Fig 5:**
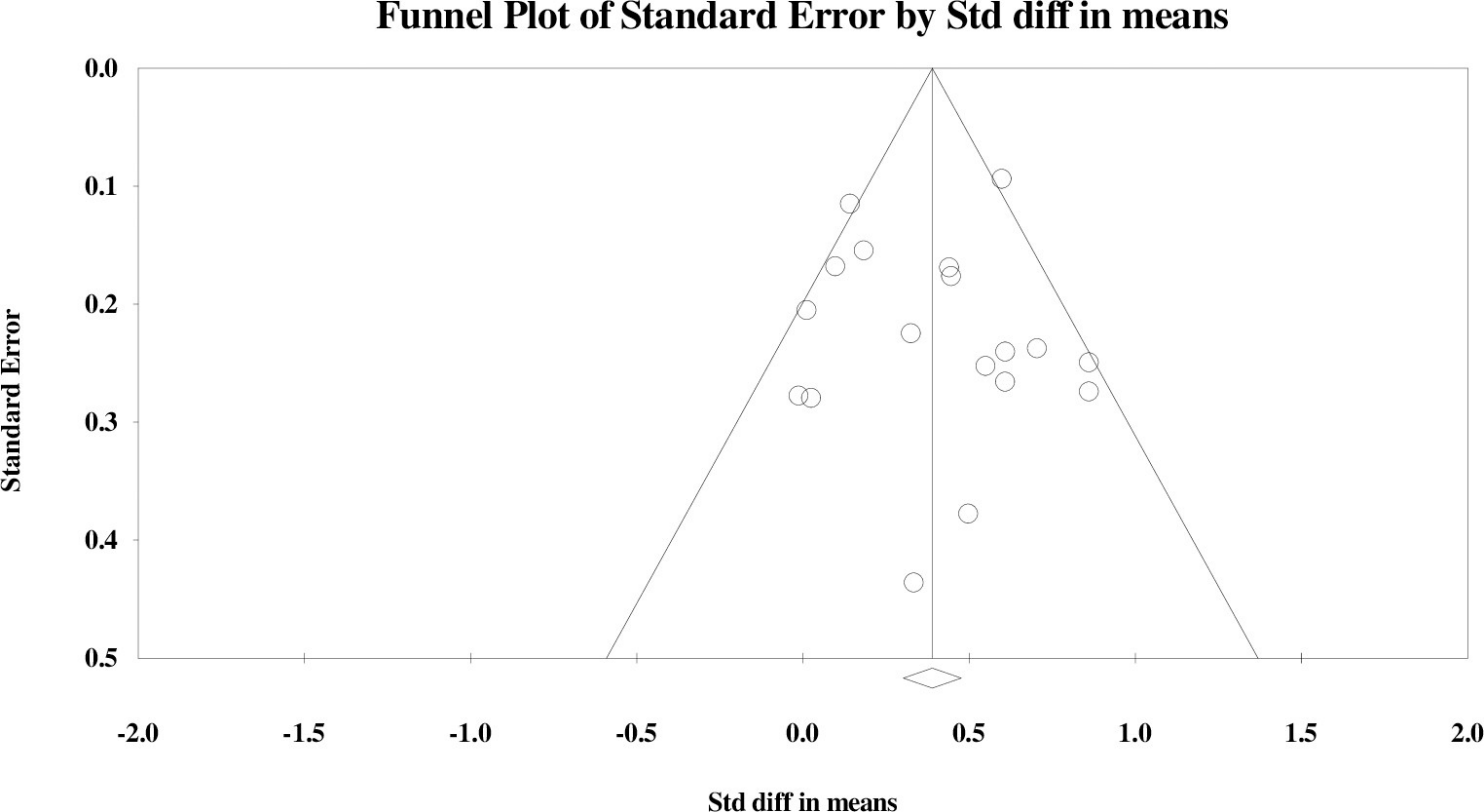
Funnel plot for publication bias for the two-arm and RCTs in the meta-analysis.

**Fig 6:**
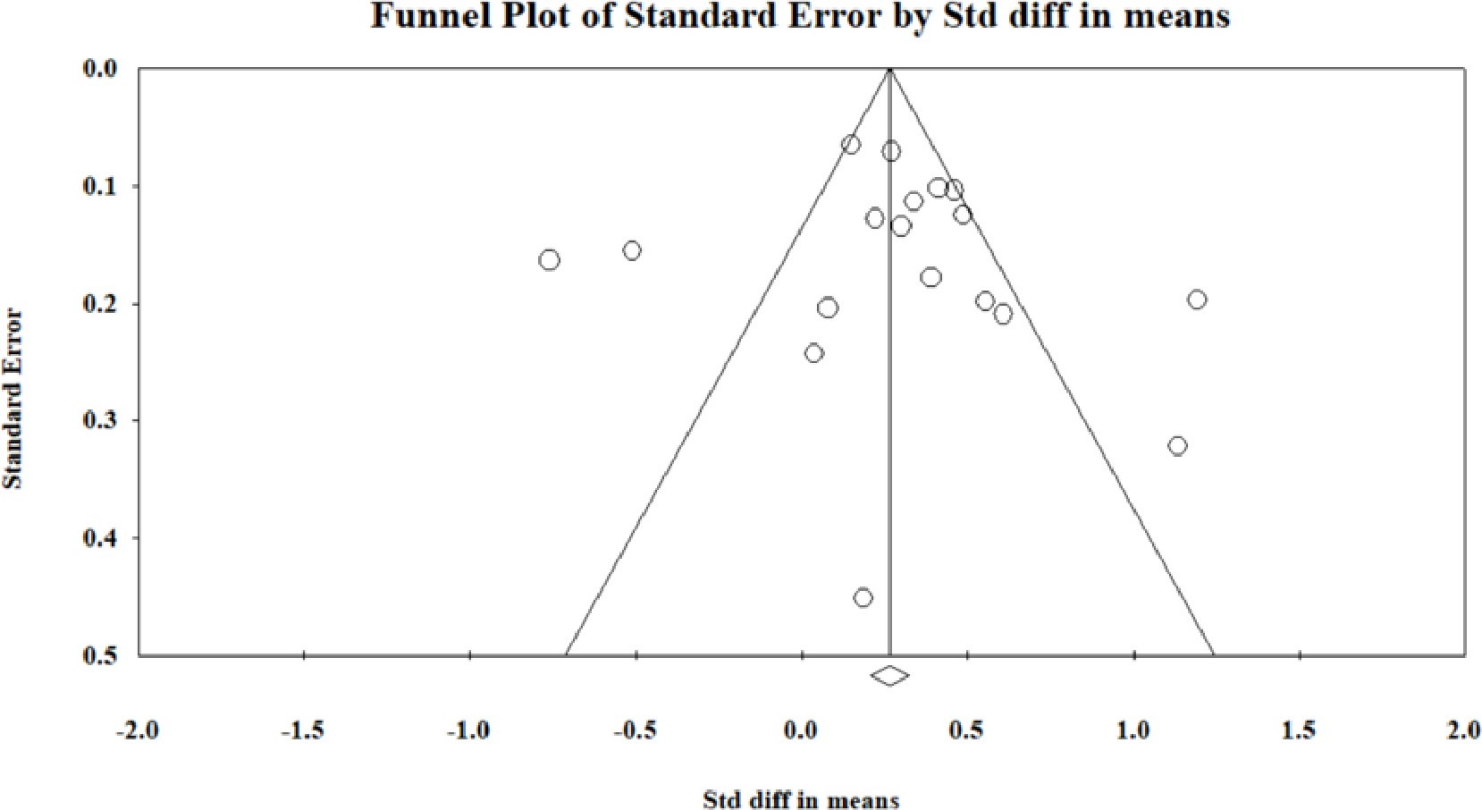
Funnel plot for publication bias of the one-arm studies in the meta-analysis.

Issues in interpretating traditional funnel plots can occur with a small number of studies, as plots can look asymmetric by chance without publication bias being present [73]; however, for completeness, they are included here (Figs 5 and 6). The funnel plots showed general symmetry, and effect sizes are seen in the lower left corner of the plot representing studies with lower effect sizes, demonstrating this meta-analysis includes studies which possibly faced publication bias due to lack of significant results (Figs 5 and 6).

The Egger’s regression test in the two-arm meta-analysis (*t* = 0.04, *p* = 0.97, intercept 0.04) and one-arm meta-analysis (*t* = 0.47, *p* = 0.64, intercept 0.67) were not significant, suggesting that the data sets are unlikely to be influenced by small sample bias [75]. A one-study-removed sensitivity analysis was run in CMA for both meta-analyses and no studies were identified as having undue influence on the magnitude of the summary effect sizes. These analyses together suggest little if any publication bias in the meta-analytic data sets.

### Moderator analyses

Moderator analyses were undertaken to investigate heterogeneity, and there were several significant findings (see Table 3). The average effect size associated with the *absence* of required home practice was larger in the two-arm studies (*d =* 0.586, *p* < .001) than that associated with studies which required home practice (*d =* 0.318, *p* < 0.001; *Q*-between = 1.370, *p* of *Q* = 0.242); this was also true for the one-arm studies (absence of required home practice: *d* = 0.426, *p* < 0.001; required home practice: *d* = 0.186, *p* = 0.102; *Q*-between = 3.820, *p* of *Q* = 0.148). In the one-arm meta-analysis the average effect size was greater in studies with mindfulness intervention delivered to clinical students as compared to preclinical students, but preclinical vs. clinical participants in two-arm studies were very similar (see Table 3). In the one-arm studies, elective curriculum (*d* = 0.482, *p* = <0.001) was more effective than mandatory curriculum (*d* = 0.075, *p* = 531, *Q* = 6.862, *p* of *Q* = 0.032); the opposite was found in the two-arm studies, but with a low number of studies using mandatory curriculum which reduces trust in the results and should be viewed with caution. Interventions with yoga produced better stress outcomes in the one-arm studies, but those without yoga produced better stress outcomes in the two-group studies.

**Table 3:** Moderator analyses.

Subgroup Analysis						
Two-arm studies	Cohen’s *d*	*k*	*p* value	95% CI	Q_between_	*p* value for *Q*
Preclinical vs	0.375***	8	<0.001	0.264–0.486	0.195	0.907
clinical students	0.347***	5	<0.001	0.188–0.505		
United States vs	0.375***	6	<0.001	0.252–0.498	0.372	0.542
international	0.370***	12	<0.001	0.256–0.484		
RCTs vs	0.356***	8	0.001	0.155–0.558	0.025	0.875
non-randomized	0.378***	10	<0.001	0.200–0.557		
At-home practice vs	0.318***	13	<0.001	0.173–0.464	1.370	0.242
no home practice	0.586***	5	<0.001	0.245–0.728		
Elective vs	0.314***	15	<0.001	0.176–0.452	7.096**	0.008
mandatory curriculum	0.602***	**3**	<0.001	0.441–0.764		
Included yoga	0.199***	**4**	0.143	-0.067–0.466	5.631	0.060
without yoga	0.438***	14	<0.001	0.298–0.578		
**One-arm studies**						
Preclinical vs	-0.021	7	0.884	-0.298–0.257	11.720**	0.008
clinical students	0.387***	6	<0.001	0.284–0.409		
United States vs	0.190	9	0.339	-0.200–0.581	1.604	0.448
international	0.289***	6	<0.001	0.191–0.388		
At-home practice vs	0.186	11	0.102	-0.037–0.409	3.820	0.148
no home practice	0.426***	4	<0.001	0.275–0.576		
Elective vs	0.482***	7	<0.001	0.232–0.733	6.862*	0.032
mandatory curriculum	0.075	8	0.531	-0.159–0.309		
Included yoga	0.491**	5	0.003	0.169–0.814	7.806*	0.050
without yoga	0.080	9	0.504	-0.155–0.315		
**Meta regressions**						
**Two-arm studies**	**Coefficient**	***k***	***p* value**	**95% CI**		
Total hours	-0.0032	15	0.832	-0.032 –+0.026		
Total no. of sessions	0.0064	15	0.247	-0.005– +0.017		
Avg duration of sessions in mins	-0.0007	15	0.658	-0.004 –+0.002		
Frequency of sessions per week	0.3044	15	0.140	-0.099 –+0.708		
Duration of intervention in wks	-0.0123	15	0.561	-0.054 –+0.029		
**One-arm studies**						
Total hours	-0.0117	11	0.051	-0.024 –+0		
Total no. of sessions	-0.0065	11	0.555	-0.028 –+0.015		
Avg duration of sessions in mins	-0.001	11	0.169	-0.003 –+0.001		
Frequency of sessions per week	0	11	0.999	-0.124 –+0		
Duration of intervention in wks	-0.059	11	0.062	-0.120 –+0.003		

*p≤.05

**p≤.01

***p≤.001

Meta-regression analyses did not produce statistically significant results for total hours, total number of sessions, average individual session duration, frequency of sessions, or duration of the entire intervention in either meta-analysis (Table 3).

## Discussion

This work presents a systematic review and meta-analysis on the effects of mindfulness interventions among medical students internationally. Significant findings include a small-to-moderate summary effect size of *d* = 0.385 compared to control groups. The absence of required home practice and less total hours in the mindfulness intervention resulted in significantly better stress outcomes. As predicted, there was wide variety in the type of mindfulness interventions, duration in weeks, duration per session, combinations of interventions, delivery methods, and effectiveness. Unexpectedly, the results of the meta-regression suggested that dosage of features of the intervention were either uncorrelated or negatively associated with the magnitudes of the effect sizes. This may partly be due to the fact that even though some interventions were very short (a day) and some long (greater than 8 weeks), the great majority of the interventions were 6–8 weeks, and as such, lack in variation in length of the interventions may have contributed to the lack of association. The data suggesting that eliminating at-home practice and reducing the hours of intervention results in improved stress outcomes may reflect the fact that medical students are exceedingly busy, and less demanding interventions may be more accessible for them. Research suggests a minimum of eight weeks is needed before changes in the brain can be visualized on MRI [76]. Alternately, it may reflect selection bias or indicate that there is an effect from simply selecting to do any mindfulness at all.

Although most of the subgroup analyses were not significant, our findings suggest that mindfulness interventions may be equally effective at reducing stress among medical students in both clinical and preclinical years, students receiving their education in the United States compared to internationally, or RCTs compared to non-randomized two-arm studies.

The meta-analytic findings suggest mindfulness training is associated with a significant, small to moderate improvement in stress among medical students compared to controls. We found a slightly higher effect size than have previous meta-analyses on mindfulness interventions, incorporating a relatively larger number of effect sizes and focusing exclusively on medical students, which more accurately reflects the influence of mindfulness on reductions in stress among this population.

The primary articles provide a rich mine of ideas for implementing mindfulness into medical schools. Mindfulness has been implemented by integrating it into the preclinical curriculum in the form of elective or mandatory courses, such as the 11-week “Embodied Health” elective at Boston University School of Medicine [48], or the “Stress Management and Resilience Training (SMART)” course at the Mayo Clinic School of Medicine, which was required of all first and second-year medical students [50]. The University of Ottawa incorporated peer-led sessions, which may reduce faculty workload [61]. If space and instructors are a limitation, distance-mediated courses were also successful, such as the “Mindful-Gym” DVD [44, 70], or the “Mindfulness Training Program” which emailed 5-minute guided meditations daily [71]. One program simply had the students come early each morning to practice 20 minutes of breath-based meditation before classes began; this could likely be offered to students in most settings [62].

There was a wide variety of activities and combinations of activities that participants engaged in during the interventions which are primed for future investigation. Moderator analyses also suggest future areas of research, such as direct comparisons of at-home vs. no home practice, session durations, program lengths, and other program and participant characteristics; clear conceptualization of moderator variables is necessary to facilitate future examination of characteristics. In addition, further studies looking at the effect of social support, [77], coping strategies [78], exercise habits [79, 80], sleep habits [81], pre-existing mental health conditions [82], and personality traits [83] would be appropriate, as all of these have been shown to individually influence medical students’ wellbeing.

There are several important limitations of this study, beginning with a relatively small number of studies included in the meta-analysis. The wide variety in the type and duration of interventions, while not unexpected, affects our ability to draw conclusions. We were unable to convert the effect size to an original metric due to the heterogeneity of outcome measures used to capture stress. Gray literature was included to the extent possible with the available databases, but some may have been missed. This also prevents a comparison of published and unpublished literature to fully address publication bias. There was limited data on the number of sessions students attended out of the total possible sessions, with only four studies in the systematic review reporting this number. Many of the studies were English-language translations, but some studies in other languages may have been missed, contributing to language and country bias. Finally, a number of potentially confounding factors were not address in the primary studies, such as if students were restricted from doing exercise besides that prescribed by the intervention, or if students had access to quiet practice space.

Most of the studies did not use random assignment, and there is need for more RCTs on medical students’ stress. Many of the studies were conducted by the researchers who created the intervention, delivered the intervention, and/or were at the same institution that created the intervention, and therefore results may exhibit unconscious bias. A majority of the studies had elective courses as the intervention, and all of the randomized studies were initiated with students volunteering for inclusion; therefore, sampling bias is likely present, as students more likely to engage in mindfulness self-selected into the studies.

The studies conducted in the United States included primarily white individuals. Further research focusing on and incorporating Blacks, Hispanics, Asians, and other non-white and underrepresented groups of medical students into research on stress and stress prevention is greatly needed. There was also particularly limited information on diverse socioeconomic groups, religious preferences, LGBTQIA2S+ orientation, or gender identity, though membership in a minority group has been shown to lead to significantly higher stress levels than in peers [84–86].

Only stress outcome measures were included. Other studies have found that mindfulness also has effects on reducing anxiety and depression [21, 23] and enhancing such variables as empathy [21, 48] and wellbeing [60, 63], but that was not assessed here.

## Conclusions

With more than half of medical students experiencing unmanaged stress, at least one in ten experiencing suicidality, and the COVID-19 pandemic exacerbating these issues, many of our physician trainees are clearly undergoing a mental health crisis. The effect of chronic stress on medical students is serious and life-threatening. This meta-analysis confirms the benefit of mindfulness in medical students and describes the wide variety of interventions that medical schools and preceptors have undertaken to address stress. Students who are educated on and given opportunities to engage with these techniques may experience less psychological and physiological symptoms of chronic stress. Further effort to incorporate mindfulness and other beneficial techniques into medical training is warranted.

## Supporting information

S1 Checklist PRISMA 2020 checklist. (DOCX)

S1 Table Details of search strategy. (DOCX)
