# Supplementary material for: The effect of mindfulness interventions on stress in medical students: A systematic review and meta-analysis
Source: PLoS One. 2023 Oct 5;18(10):e0286387. doi: 10.1371/journal.pone.0286387 (PMC10553303; doi:10.1371/journal.pone.0286387)
Supplement: S1 Table — (DOCX) [file pone.0286387.s002.docx]

**Supplemental Information**

**Table 1 -** Details of search strategy

| **Database or search technique** | **Date searched** | **Search terms** | **N Results** | **N Kept for further review** |
| --- | --- | --- | --- | --- |
| EBSCOhost (22 databases)* | Feb 3, 2022 | Mindfulness + medical students (Boolean)  Mindfulness [TITLE] + medical students (Boolean) | 541  206 | 89 |
|  | May 4, 2022 | Medical students + stress + yoga [Titles] Boolean  Medical students + stress + yoga [Abstract] Boolean | 4  21 | 3  3 |
| EBSCO Discovery | Feb 4, 2022 | Selected categories (5): Complementary & Alternative Medicine; Health & Medicine; Nursing & Allied Health; Psychology; and Public Health  Mindfulness + medical students (Boolean)  Mindfulness [TITLE] + medical students (Boolean)  Advanced search: English only filter | 858  423  300 | 19 |
|  | May 4, 2022 | Selected categories (7): Complementary & Alternative Medicine; Health & Medicine; Nursing & Allied Health; PT & OT; Psychology; Public Health; Social Sciences & Humanities  Yoga [TITLE] + medical students (Boolean)  Advanced search: English only filter | 22 | 1 |
| PubMed | Feb 5, 2022 | (Mindfulness) AND (medical students)  (mindfulness[MeSH Terms]) AND (medical students[MeSH Terms])  (mindfulness[Title/Abstract]) AND (medical students[Title/Abstract])  (meditation[Title/Abstract]) AND (medical students[Title/Abstract]) | 586  75  130  60 | 17  21  13 |
|  | May 4, 2022 | (mindfulness[MeSH Terms]) AND (medical students[MeSH Terms])  (yoga[Title/Abstract]) AND (medical students[Title/Abstract]) | 14  33 | 0 |
| Publication Finder | Feb 20, 2022 | Journal of Mind and Medical Sciences  Key words: mindfulness, medical students  International Journal of Medical Students  Key word: mindfulness  Mindfulness  Key word: medical students  Mind, Brain, and Education  Key words: mindfulness, medical students  Medical Education | 41  2  499  16  101 | 0  0  7  0  3 |
|  | May 4, 2022 | Journal of Mind and Medical Sciences  Key words: yoga, medical students  International Journal of Medical Students  Key word: yoga  Mindfulness, Key word: yoga  Mind, Brain, and Education  Key words: yoga, medical students  Medical Education  Key words: yoga | 2  0  181  1  12 | 0  0  0  0  0 |
| Trip | Feb 20, 2022 | PICO search: P: medical students, I: mindfulness  P: medical students, I: meditation  P: medical students, I: yoga | 36  7  8 | 5  0  7 |
|  | May 4, 2022 | P: medical students, I: yoga | 8 | 0 |
| ClinicalTrials.gov | Feb 12, 2022 | Mindfulness + medical students  Meditation + medical students; Completed | 10  4 | 0 |
| ISRCTN registry | Feb 12, 2022 | Condition Category: Mental & Behavioral Disorders  Text: Mindfulness; Condition: Stress  Intervention: Mindfulness; Inclusion: Medical students  Intervention: Meditation; Inclusion: Medical students | 26  1  0 | 1  0  0 |
| SveMed+ | Feb 20, 2022 | MeSH terms: mindfulness, medical students | 26 | 0 |
| Education Full Text | Feb 23, 2022 | Mindfulness, medical students (any field)  Meditation, medical students (any field) | 14  6 | 3  1 |
| Education Index Retrospective (dates available: 1923-1983) | Feb 23, 2022 | Mindfulness, medical students (any field)  Meditation, medical students (any field) | 0  0 | 0 |
| Web of Science | Feb 23, 2022 | Mindfulness, medical students (title, abstract, key words) | 339 | 20 |
| Sociological Abstracts | Feb 23, 2022 | Mindfulness and medical students (abstract)  Meditation and medical students (abstract) | 1  1 | 0  0 |
| Hand search | Feb 5, 2022 | WMJ Vol 114 Issue 3: Creating a Culture of Mindfulness in Medicine |  | 1 |
|  | May 4, 2022 | Saoji editorial |  | 1 |

*Databases in EBSCOhost (alphabetically): Academic Search Elite, AHFS Consumer Medication Information, Alt HealthWatch, APA PsycInfo, Business Source Elite, CINAHL, eBook Collection (EBSCOhost), ERIC, Funk & Wagnalls New World Encyclopedia, GreenFILE, Health Source - Consumer Edition, Health Source: Nursing/Academic Edition, Library, Information Science & Technology Abstracts, MAS Reference eBook Collection, MAS Ultra - School Edition, MEDLINE, Military & Government Collection, Newspaper Source, Primary Search, Primary Search Reference eBook Collection, OpenDissertations, Regional Business News
